# Bone defects in knee revision arthroplasty—a therapy-oriented classification

**DOI:** 10.1007/s00402-025-05759-2

**Published:** 2025-03-29

**Authors:** Max Jaenisch, Elias Hoven, Martin Gathen, Frank Sebastian Fröschen, Adnan Kasapovic, Charlotte Rommelspacher, Andreas Christian Strauß, Dieter Christian Wirtz

**Affiliations:** Department of Orthopedics and Trauma Surgery, University Hospital Bonn, Venusberg Campus 1, 53127 Bonn, Germany

**Keywords:** Knee revision arthroplasty, AORI, Defect classification, Ligamentary instability, Constrainment, Reliability, Reproducability

## Abstract

**Introduction:**

The complex field of revision knee arthroplasty displays a lack of standardized, intuitive pre- and intraoperative assessment of bone defects. In clinical practice revision surgeries are a common sight presenting with increasingly complex cases of bone loss and ligamentary dysfunction. To address these issues the Knee Defect Classification (KDC) is introduced to offer a reliable, reproducible and an intuitive classification system with a clear therapeutic guideline.

**Materials and methods:**

Reliability was evaluated through comparison between intraoperative findings and preoperative gradings of 218 patients. To account for reproducibility inter- and intra-rater agreement was assessed.

**Results:**

The mean agreement between intraoperative and radiographic grading of femoral and tibial defects was evaluated with a Cohens kappa of 0.79, representing a good agreement. For interrater reliability a mean Fleiss kappa of 0.71 could be evaluated for femoral and tibial defects falling into the good agreement range. In the subgroup analysis femoral defects presented with a Fleiss kappa of 0.652 ± 0.026 (good agreement), while tibial defects presented with a Fleiss kappa of 0.768 ± 0.024 (good agreement). For intrarater reliability a mean Cohens kappa was evaluated at 0.78 indicating good agreement.

**Conclusion:**

The KDC is a reliable and reproducible classification system. Through its structured design it facilitates intuitive use and allows for consistent preoperative planning and intraoperative guidance. A therapeutic algorithm is provided based on a review of the literature in combination with expert opinion.

## Introduction

Total knee arthroplasty (TKA) is the second most common joint replacement surgery in Europe and intervention numbers are continuously on the rise. Due to the increased life expectancy of an aging population and the increase in arthroplasties for younger patients, revision rates are expected to rise as well. [1, 2] In clinical practice even re-revision surgeries are a common sight presenting with increasingly complex cases of bone loss and ligamentary dysfunction.

Recently, the same group successfully introduced an innovative classification system of bone defects of the hip with the Acetabular Defect Classification (ADC) and the Femoral Defect Classification (FDC). [3, 4] In this publication a similar classification system for osseous and ligamentary defects of the knee will be introduced.

Proper reconstruction of the joint line, sufficient primary stability and adequate constrainment of the components, dictated by the ligamentary dysfunction, are the main pillars of successful revision knee arthroplasty. The aim is to achieve full weight-bearing and sufficient range of motion to enable proper mobilization of the patient. In most cases, functional outcome and revision-free survival are inferior to those of primary knee arthroplasty. [5]

The principle of zonal fixation introduced by Morgan-Jones et al. describes three anatomical zones of the femur and tibia which can be used to support the implant. Fixation of the component can be achieved at the level of the epiphysis, the metaphysis and the diaphysis, through varying components. [6] Bone loss in these anatomical zones may hinder stable fixation and pose a great challenge to surgeons in revision total knee arthroplasty (rTKA). Modern revision arthroplasty systems included a multitude of different components to aid primary stable fixation and long term osseointegration. These included modular stems, augments, metaphyseal sleeves, cones and more. [7] Due to the complexity of this field of practice detailed preoperative planning and reliable defect recognition are essential.

Throughout the clinical evolution of rTKA, 16 classification systems have been introduced to aid the decision process pre- and intraoperatively and enable a dedicated scientific conversation around common defect morphologies. [8–24] While some classification systems focus on the morphology of the bone defects, others are based on a quantitative assessment of the defect size. The most commonly used classification system is the AORI (Anderson Orthopaedic Research Institute) classification by Engh et al. introduced in 1997. [14] While it provides a detailed defect description based on size, location and soft tissue involvement, it fails to include the diaphysis and does not deliver a comprehensive therapeutic algorithm. Due to the focus on morphological defect definitions, rater reliability is not satisfactory. [25] Recently, the group around Scuderi and Mont proposed a new classification system specifically focusing on the metaphyseal and diaphyseal bone loss due to the revision of stemmed total knee arthroplasty. They however neglect the ligamentary function and cater to a specific subgroup of rTKA and therefore do not offer a holistic approach. [24] Table 1 presents an overview of available classification systems in comparison to the KDC.

**Table 1:** Comparison of established knee defect classification systems to the KDC

Defect classification	Evaluation of defect containment	Modern therapeutic algorithm	Complete ligamentary evaluation	Diaphyseal inclusion
KDC	Yes	Yes	Yes	Yes
AORI [14]	None	None	Partially	No
Stambough et al. [20]	Yes	Yes	Partially	No
Rosso et al. [22]	None	Yes	Partially	No
Belt et al. [23]	None	None	None	Yes
Scuderi et al. [24]	None	None	None	Yes

To authors’ knowledge, no classification system is available that combines all three zones (epiphysis, metaphysis and diaphysis) and an assessment of ligamentary function in combination with a clear therapeutic algorithm.

The aim of this publication is to provide an intuitive classification system for the pre- and intraoperative evaluation of osseous and ligamentary defects of the knee joint which is based on state-of-the-art treatment options. Reliability will be evaluated through comparison between intraoperative findings and preoperative gradings. To account for reproducibility inter- and intra-rater agreement will be assessed. The four main categories (1–4) determine the location and extent of the encountered defect for the femur and tibia respectively. Additional subcategories (A-C) characterize the impairment of the ligamentary structures.

We hypothesized that the KDC is a reliable and reproducible classification system. It provides a reliable defect estimation preoperatively which may be applied in the planning process. A therapeutic algorithm is supplied to help with implant choice and additional interventions according to current literature and expert opinion. The proposed Knee Defect Classification (KDC) joins the recently introduced Acetabular Defect Classification (ADC) and Femoral Defect Classification (FDC) to further complete an integrated and didactically similar classification system for the complex field of revision arthroplasty.

## Materials and methods

### Study design and patients

A single center cohort study based on retrospectively collected data of patients that underwent rTKA between 2015 and 2021 was conducted. 252 consecutive patients who received rTKA for various reasons were identified. Exclusion criteria were incomplete preoperative radiographic documentation or insufficient quality of radiographs and incomplete intraoperative documentation unconducive of reliable grading. In addition, characteristics including age, sex, date of revision and implant inserted were collected. No other patient characteristic or clinical data were collected as they are not subject of this evaluation. Our study exclusively evaluated defect configuration on radiographs and intraoperatively. 18 patients were excluded because no digital copies of preoperative radiographs could be retrieved or because radiographs were of insufficient quality. Furthermore, 16 patients were excluded because intraoperative data did not match the defined requirements (see intraoperative evaluation). After exclusion, 218 patients were evaluated.

### Intraoperative evaluation

Prior to grading, the surgical documentation was screened and information concerning the extend of defect morphology and size, as well as additional information (e.g., implants used/removed and/or use of augmentation) were recorded. Exclusion criteria were lack of description for the exact defect location and extend, missing measurement of defect size and incomplete radiographic documentation or radiographs of insufficient quality.

Retrospectively collected intraoperative data was graded according to KDC by the first author, who was not involved in the collection of the data and therefore had no prior knowledge of the corresponding radiographs for each case.

### Radiographic evaluation

Preoperative radiographs included an A.P. and a lateral view of the knee joint. Radiographic analysis was carried out using IMPAX EE (Agfa HealthCare GmbH, Bonn, Germany). All radiographs were anonymized. The first author performed the preoperative radiographic grading for the whole collective. 5 experienced orthopaedic surgeons in the field of revision arthroplasty rated a randomized sample of 80 patients. Each rater received a teaching session consisting of supervised evaluation of 10 random cases. The 10 sample cases were not part of the scored evaluation. Each rater received a scoring sheet. The raters had no prior knowledge of the KDC, nor were they involved in the conception of the classification system. A wash out period of 2 weeks was set between rating sessions to prevent a bias due to memorization of the defects. Radiographs were relabelled and randomized prior to the second evaluation.

### Classification system

When discussing a stable fixation of a revision knee arthroplasty the epiphysis, metaphysis and diaphysis must be regarded. The implant must be fixed in at least 2 of the 3 main zones of the femur and tibia. The KDC adheres to the integrity of these structures and to the established principle of zonal fixation. [6] The four main categories (1–4) determine the location and extent of the encountered defect for the femur and tibia respectively. Additional subcategories (A-C) characterize the impairment of the ligamentary structures.

#### Type 1 defects

**Fig. 1:**
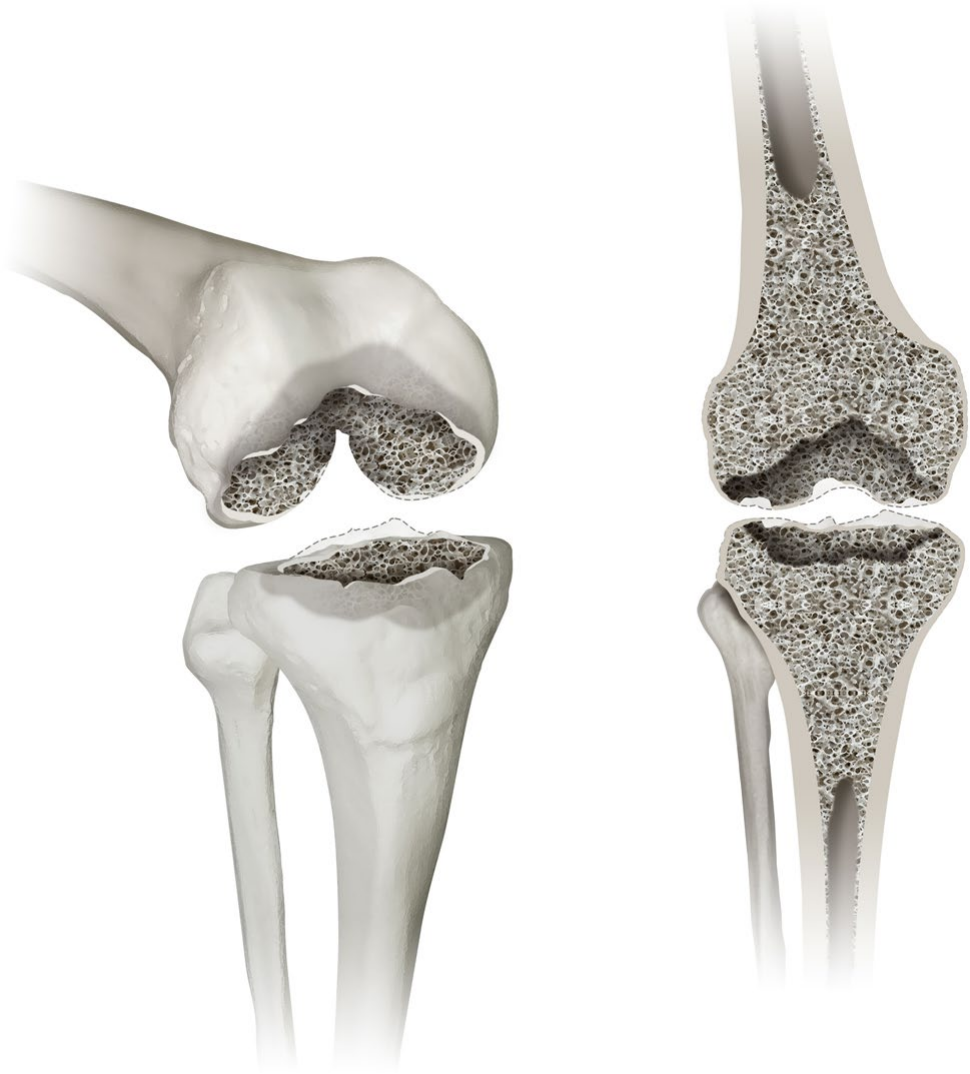
displays a type 1 defect of the Knee Defect Classification (KDC). Type 1 is characterised by isolated defects of the femoral and tibial epiphyseal bone stock. The left image shows a 3D oblique view and the right image a frontal view

Type 1 includes all isolated defects of the femoral and tibial epiphysis. Such defects may be encountered during revision surgery of a standard total knee replacement, which presented with loosening and did not create a large bone defect during removal. An illustration of Type 1 is displayed in Fig. 1.

#### Type 2 defects

**Fig. 2:**
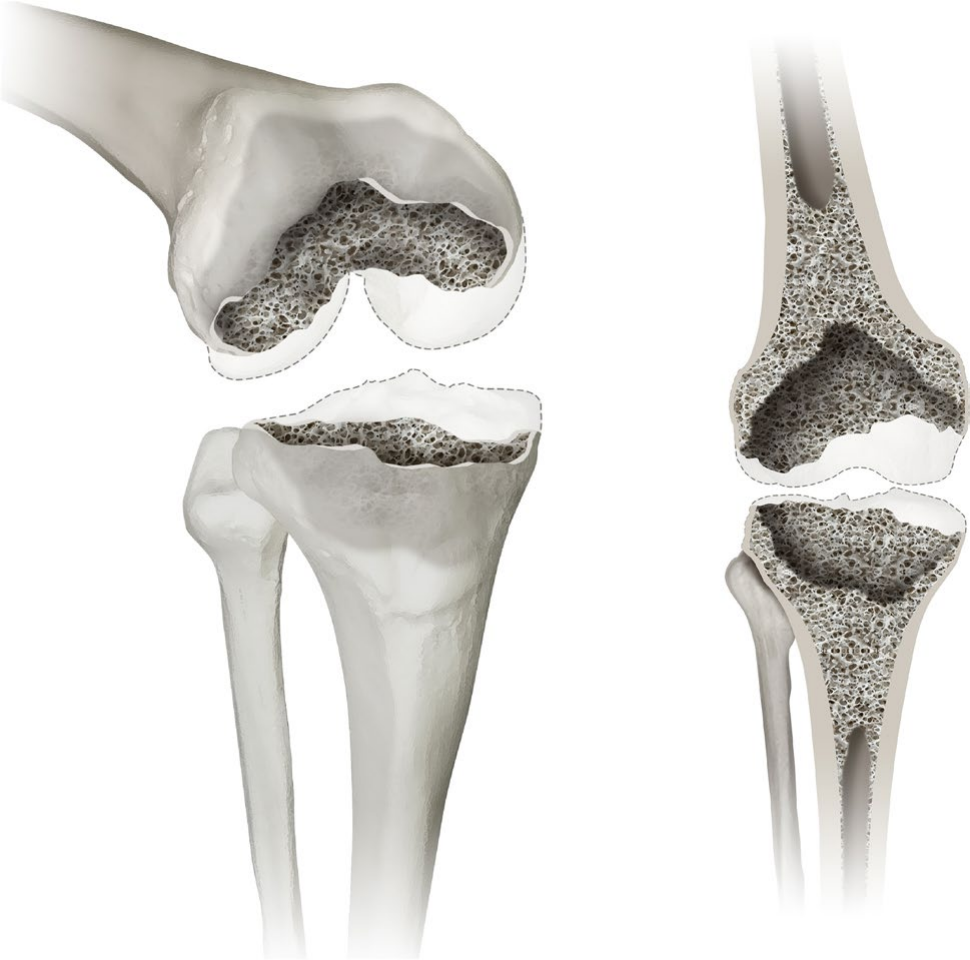
displays a type 2 defect of the Knee Defect Classification (KDC). In type 2 defects the surgeon encounters a cancellous depletion of the metaphysis and/or a contained defect of the metaphyseal cortical bone (less than 50% of the circumference), which is displayed in an 3D oblique (left) and a frontal view (right)

Type 2 presents a cancellous depletion of the metaphysis and/or a contained defect of the metaphyseal cortical bone (less than 50% of the circumference). These defects most commonly occur when a well-fixed total knee arthroplasty is removed (e.g., due to an acute periprosthetic infection) or prolonged endoprosthetic loosening with more profound bone loss. Figure 2 illustrates a type 2 defect in different views.

#### Type 3 defects

**Fig. 3:**
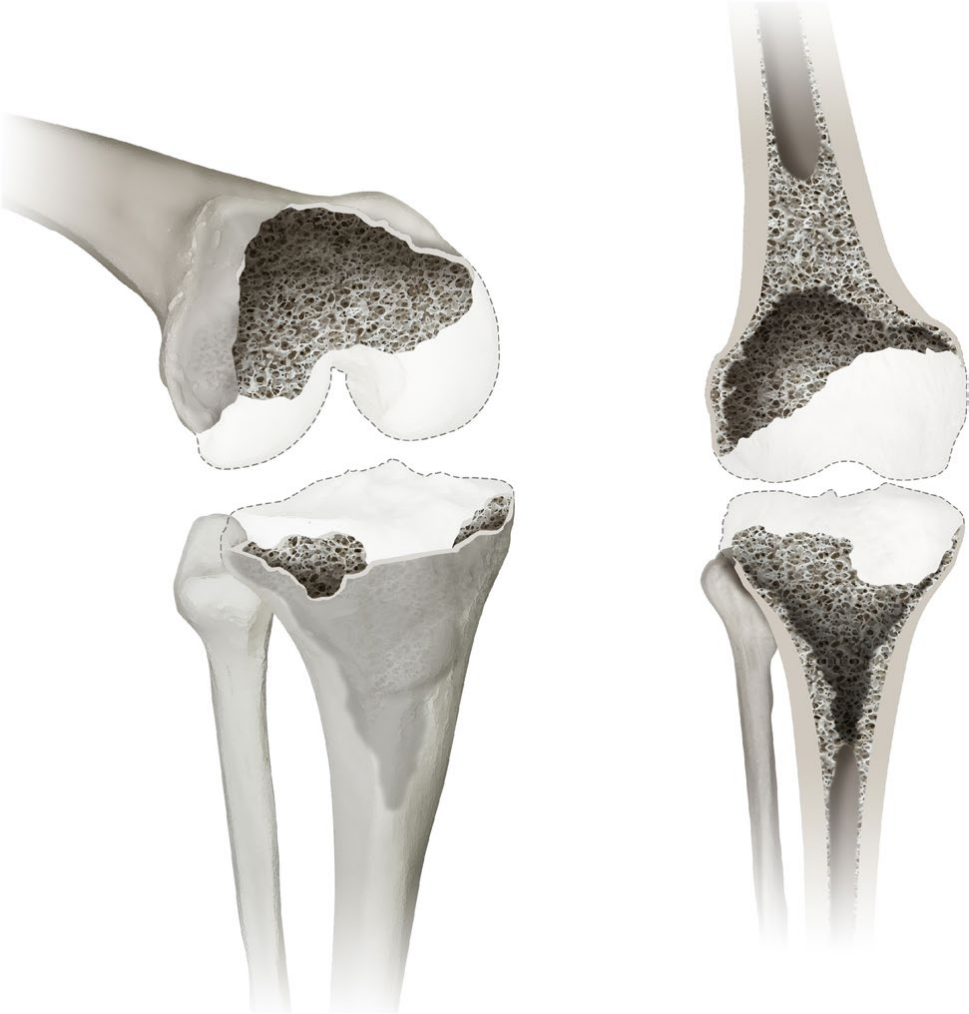
displays a type 3 defect of the Knee Defect Classification (KDC) in a 3D oblique view (left) and a frontal view (right). While type 3 defects still present with cancellous depletion, the cortical defect of the metaphysis is considered as uncontained (more than 50% of the circumference) in contrast to a contained defect in type 2

Type 3 defects are limited to the metaphyseal bone similar to type 2 defects. Cancellous depletion is also encountered, but the cortical defect of the metaphysis is considered as uncontained (more than 50% of the circumference). These defects are generally caused by either removal of a cemented or cementless total knee arthroplasty with stems or a standard knee arthroplasty but greatly reduced bone quality due to e.g., osteoporosis and/or rheumatic diseases. Figure 3 details a graphic reproduction of the type 3 defect.

#### Type 4 defects

**Fig. 4:**
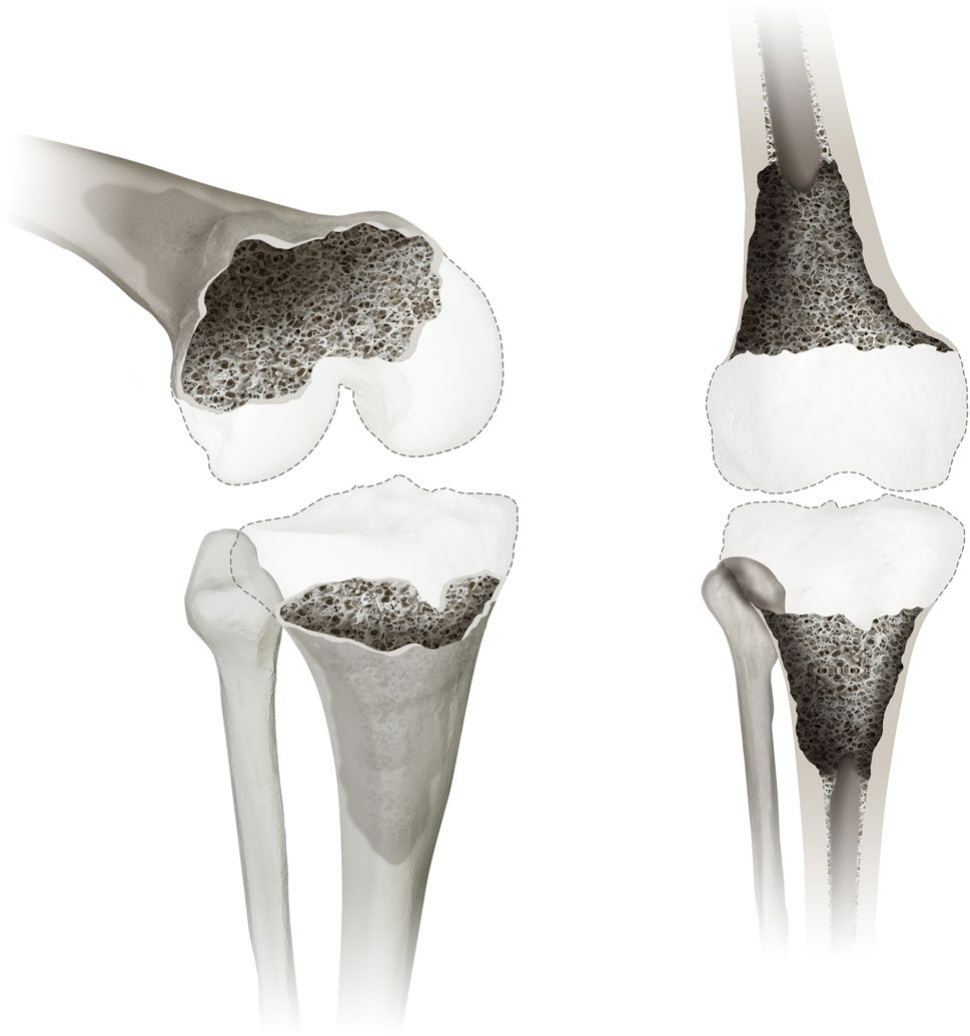
displays a type 4 defect of the Knee Defect Classification (KDC). Type 4 describes the most severe bone loss in this classification affects the diaphysis of the femur and/or tibia. A 3D oblique view (left) and a frontal view (right) are provided

Type 4 represents the most severe bone defect in this classification and in addition to the aforementioned types also affects the diaphysis of the femur and tibia. These defects may be caused by extensive loosening of femoral or tibial stems or even periprosthetic fractures. Figure 4 shows a type 4 defect from two different perspectives.

#### Subcategories A-C

Proper function and the needed type of constrainment of a revision knee arthroplasty are greatly determined by the integrity of the ligamentary structures of the joint including the medial and lateral collateral ligament and the patellar bone with the patellar- and quadriceps tendon. To enable holistic description of the pathology, a ligamentary assessment has been added to the KDC through the application of subcategories. A is considered a stable joint with a proper extension mechanism. Depending on the type of implant removed and the soft tissue damage caused by previous operations, scarring and infection, this is rarely the case. B describes a ligamentary instability which can be in a single plane but also multidirectional. The extension mechanism is not affected. For C, an insufficiency of the extensor mechanism is encountered. Other ligamentary defects resulting in a multidirectional instability may be encountered.

### Statistical analysis

For the statistical analysis IBM SPSS Statistics 1.0.0.1131 (IBM Inc., Armonk, New York, USA) was used. The level of significance was set at p < 0.05. The confidence interval has been set at 95%. To evaluate interrater reliability for the comparison of ordered categorical data with more than 2 raters Fleiss kappa was used. To summarize femoral and tibial defects means of the individual, Cohens kappa values have been calculated. Cohens kappa was applied to analyze intra-rater reliability between each rater. The results are the mean kappa values of all raters. Interpretation of kappa values was carried out through the application of the agreement scale described by Landis and Koch [26]. Kappa values exceeding 0.80 indicate excellent agreement, between 0.61 and 0.8 indicate good agreement, between 0.41 to 0.60 indicate moderate agreement, between 0.21 and 0.4 indicate fair agreement and between 0.20 and below indicates poor agreement.

## Results

**Fig. 5:**
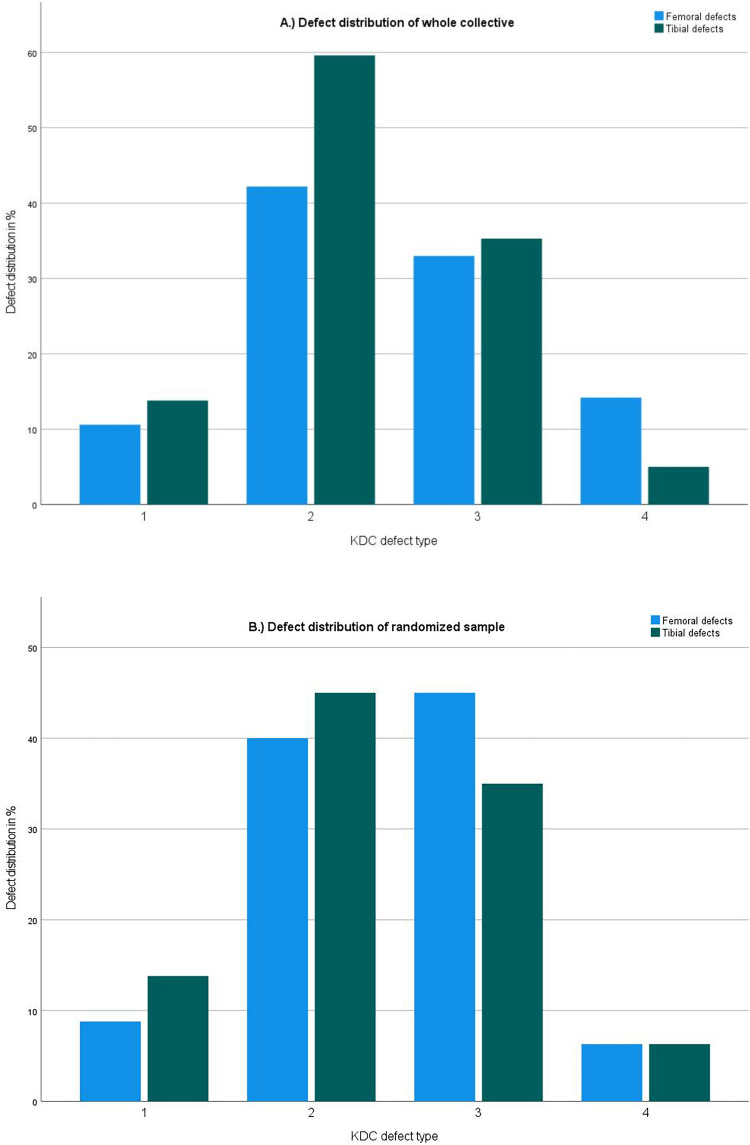
To allow for a clinically relevant evaluation of the reproducibility of the KDC all described defects need to be included in the analysis. When evaluating the distribution of defect types in the whole collective and the randomized sample all defects were included. The distribution matched the clinical reality in the authors practice. **a** Illustration of the distribution of KDC defect types in the whole collective (n = 218); y-axis: percent out of all cases, x-axis: KDC Defect type 1–4; femoral defects (blue); tibial defects (green). **b** Illustration of the distribution of KDC defect types in the randomized sample (n = 80); y-axis: percent out of all cases, x-axis: KDC Defect type 1–4; femoral defects (blue); tibial defects (green)

After exclusion, 218 patients were evaluated. Each defect type could be identified in the collective. The distribution of defect types in the intraoperative evaluation of the whole collective and in the radiographic evaluation of the randomized sample reflected clinical reality in the authors’ institution. A graphical illustration is shown in Fig. 5.

### Reliability

The mean agreement between intraoperative and radiographic grading of femoral and tibial defects was evaluated with a Cohens kappa of 0.79**,** representing a good agreement. In the subgroup analysis femoral defects presented with a Cohens kappa of 0.753 ± 0.038 (good agreement), while tibial defects presented with a Cohens kappa of 0.830 ± 0.033 (excellent agreement).

### Reproducibility

For interrater reliability a mean Fleiss kappa of 0.71 could be evaluated for femoral and tibial defects falling into the good agreement range. In the subgroup analysis femoral defects presented with a Fleiss kappa of 0.652 ± 0.026 (good agreement), while tibial defects presented with a Fleiss kappa of 0.768 ± 0.024 (good agreement).

**Fig. 6:**
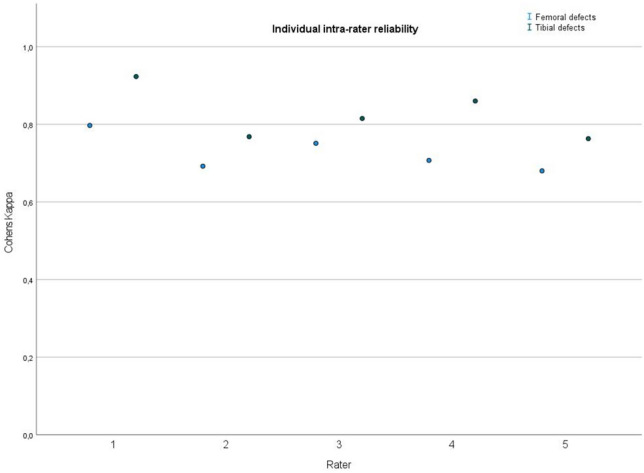
Illustration of the individual results (Cohens kappa) of intra-rater reliability decided in femoral (blue dot) and tibial (green dot) evaluation. All individual raters displayed good to excellent agreement between themselves at different time points. Raters consistently scored a lower Cohens kappa in the evaluation of femoral defects when compared to the evaluation of tibial defects; y-axis: Cohens kappa, x-axis: individual rater

For intrarater reliability a mean Cohens kappa was evaluated at 0.78 indicating good agreement. In the subgroup analysis femoral defects presented with a mean Cohens kappa of 0.725 (good agreement), while tibial defects presented with a mean Cohens kappa of 0.826 (excellent agreement). Individual results for each rater are displayed in Fig. 6. Throughout the whole evaluation, femoral defects showed a lower agreement than tibial defects.

## Discussion

Failure of TKA can cause severe peri-prosthetic bone loss and compromise the ligamentary stability and function. To aid in the successful execution of rTKA, detailed preoperative planning and reliable defect recognition are essential. Multiple classification systems have been introduced since the late 1980s. Many of the early classification systems focused on appearance, morphology and severity of the defect and are rarely used today. [8, 9, 12, 18] Others attempted a type-distinction through size of the defect in varying methods of measurement. [10, 11, 13, 15–17] The group of Malefijt introduced the concept of contained vs. uncontained defects to the description of periprosthetic bone defects of the knee, which remains relevant today. [13] The most used classification system in current literature is the AORI classification introduced by Engh et al. It presented a vast improvement over existing systems and included defect size, location, and even soft tissue involvement. AORI type I defects are minor osseous deficiencies close to the joint line. Type II defects are more extensive defects of the central and peripheral metaphyseal bone with an intact or partially absent cortical rim involving one condyle (IIA) or both condyles (IIB) of the femur. In type II defects, the origins and insertions of the collateral ligaments are preserved. AORI type III involves extensive loss of metaphyseal bone with associated substantial deficiency of cortical bone and may include a defect of the collateral ligamentary attachments. [14] To improve upon the AORI classification various modifications have been published. The group of Stambough et al. added containment of the defect to the system and Rosso et al. further subcategorized the AORI classification by adding the modifier "bone quality" (G-good, S-sclerotic, O-osteoporotic). [20, 22] A recent introduction of a classification system for rTKA was contributed by Belt et al. Their systems featured the involvement of the diaphyseal bone for the first time. [23] The latest addition to the classification of rTKA has been introduced by Scuderi et al. especially focusing on the revision of stemmed TKA paying special attention to the metaphyseal and diaphyseal bone. [24] However, to the authors’ knowledge, no classification system includes and rates all relevant aspects in the planning and the execution of rTKA while still maintaining ease-of-use and clinical relevance. To improve upon existing approaches, the KDC especially focuses on the differences between defects that directly translate to implant choice. Thereby it maintains an ease-of-use without overcomplicating defect descriptions. Adhering to the principle of zonal fixation, all main aspects of stability (epiphysis, metaphysis, and diaphysis) are included. By adding ‘ligamentary function’ all aspects of implant choice are considered.

The most important criterion for a classification system in the authors’ opinion is reliability. The surgeon has to be certain that the defect he or she rates preoperatively will be encountered in the operation with a high probability. In this publication, the evaluation of the agreement between preoperative grading and intraoperative finding showed a good result. The calculated mean kappa of 0.79 falls in the good agreement range and we therefore concluded that the KDC offers reliable preoperative grading. The evaluation of the femoral defects rated with a lower agreement (0.753 ± 0.038, good agreement) when compared to the tibial defects (0.830 ± 0.033, excellent agreement). This is a trend that was encountered throughout the whole evaluation and may be caused by the more complex configuration of the femoral bone and the higher likelihood of the presence of radiopaque components obscuring especially the femoral condyles. The encountered problems in the femoral assessment of radiographs have been reported in the literature before. Especially smaller bone gaps (AORI I and IIa) may be overlooked according to an evaluation by Iamaguchi et al. [27] In a recent systematic review of different knee defect classifications, a similar result has been observed with a significantly lower performance in the reliability of grading femoral defects compared to tibial defects. [25] In regard to reliability, the KDC compares favorably to the established AORI classification. In the literature analysis, 3 different studies could be identified that evaluated reliability (agreement between preoperative grading and intraoperative findings) of the AORI classification. They reported an average kappa value for femoral bone loss at 0.38 (0.27–0.50) and for tibial bone loss at 0.76 (0.63–1). [25, 28–30] The authors contribute the better results of the KDC to the structured design and the focused assessment of clinically relevant defects. Another aspect of reliability is the application of the same classification system to the same radiographs by different raters. The so-called interrater reliability is an established parameter in literature and displayed good results throughout all 5 raters in this evaluation. The raters achieved a mean kappa of 0.71 (femoral: 0.652 ± 0.026, tibial: 0.768 ± 0.024), falling into the good agreement range. Finally, a rater should ideally choose the same grading for the same defect at different times, which is assessed by evaluating intrarater reliability. The evaluation of intrarater reliability for the KDC resulted in a mean kappa of 0.78 (femoral: 0.725, tibial 0.826) accounting for good agreement. While evidence on interrater and intrarater reliability is limited, the classification of Scuderi et al. showed comparable results (good agreement for inter- and intrarater reliability) using a similar evaluation progress. [24] The classification system introduced by Belt et al. has been reported with an interrater reliability between 0.48 and 0.97 and an intrarater reliability between 0.55 and 0.99. Again, as seen in the aforementioned studies and our own evaluation, femoral defects presented with a lower agreement than tibial defects, especially at the epiphysis. [18]

In the following paragraphs, the therapeutic recommendations according to defect type will be discussed. The therapeutic algorithm was established through expert opinion in combination with a thorough review of the current literature and derived from the therapeutic procedure of the authors’ own clinical experience. Treatment options for the specific defects will be discussed for each zone separately. Adhering to the principle of zonal fixation, stable fixation in at least one additional zone is necessary to achieve long term implant stability. [6] Therefore, it is implied that the planning of the final implant choice needs to include at least two zones. The four main categories ascend in severity and are treated in a hierarchical order. If a diaphyseal defect (type 4) presents without a metaphyseal defect the metaphysis should be treated according to type 2/3).

**Fig. 7:**
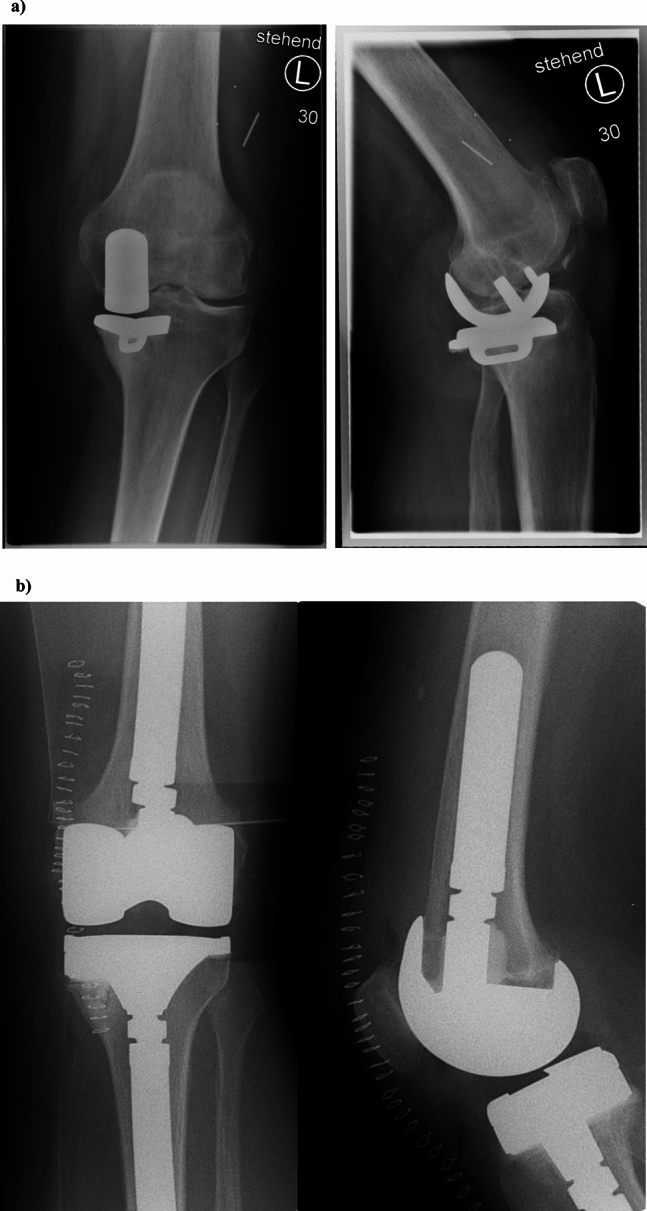
Case 1 shows a 71-year old female patient with a loosening of a medial hemiprosthesis with a consecutive bony KDC 2 defect of the tibia and medial instability KDC B. **a** Preoperative x-rays anterior–posterior view on the left and lateral view on the right. A mal rotated hemiprosthesis with loosening can be seen. Clinically the patient also displays a medial instability with a high likelihood for bony as well as ligamentary damage (KDC B). **b** Postoperative x-rays anterior–posterior view on the left and lateral view on the right. The tibial KDC 2 bone defect has been treated with a block augment (10 mm) creating sufficient force transmission and rotational stability in addition to a modular un-cemented stem. A semi-constrained implant was chose due to remaining instability after anatomic reconstruction of the joint line

**Fig. 8:**
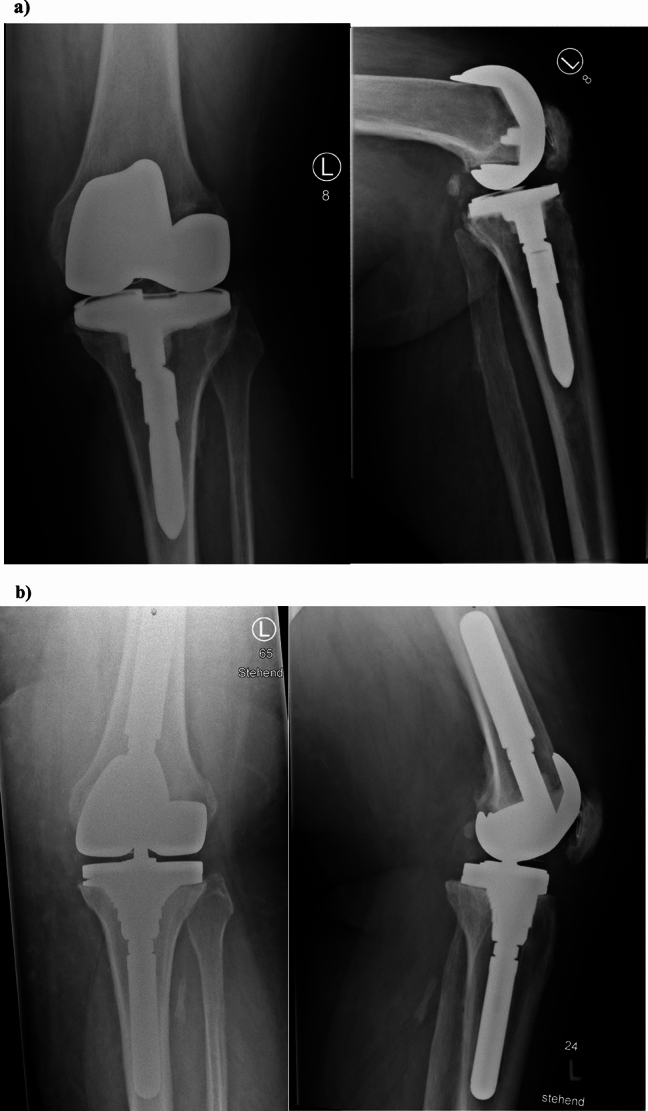
Case 2 shows a morbidly obese, 52 year-old male patient with a multidirectional instability KDC B and aseptic loosening of the femoral and tibial component. **a** Properative x-rays anterior–posterior view on the left, lateral view on the right. Unconstrained, cruciate-retaining prosthesis with a tibial modular un-cemented stem. Radiolucent lines can be observed on the femur and around the tibial stem. **b** Postoperative x-rays anterior–posterior view on the left and lateral view on the right. After removal of the implant in this single-stage exchange the femur displayed a KDC 1 defect. The small epiphyseal defects could be augmented with PMMA. The removal of the tibial component resulted in a KDC 2 defect which was treated with a metaphyseal sleeve and an un-cemented modular stem. Due to the persistent instability after anatomic reconstruction of the joint line (KDC B) a rotating-hinge prosthesis was chosen

Type 1 defects affect the epiphyseal bone of both the femur and tibia. These defects will be present in a multitude of rTKA cases and can be addressed through different means. Smaller uncontained and contained defects up to 5 mm can be augmented with PMMA or bone allograft (see Fig. 8). In larger defects, biomechanical stability tends to be lower compared to metallic means of augmentation and is not recommended by the authors. [31] Larger defects and especially uncontained defects should receive a metallic augmentation to enable proper force transmission to the remaining host bone (see Fig. 7). Usually, block augments are attached to the femoral or tibial component prior to implantation. These type of block augments can be used for tibial and femoral defects up to 15–20 mm and therefore can be used in type 2 defects extending in the metaphyseal bone as well (see Fig. 7). [32, 33] The conversion of uneven peripheral defects in block shaped defects creates an improved biomechanical stability. [32]

In type 2 defects, a cancellous depletion of the metaphysis and/or a contained defect of the metaphyseal cortical bone is present. In addition to metallic block augments, as mentioned above, metallic sleeves offer a new alternative technique of augmentation (see Fig. 8) with promising intermediate-term results. [34–36] Sleeves are implant-specific and attach to the component prior to implantation. Cancellous depletion and limited cortical defects can be augmented and the macroporous configuration allows for host bone ingrowth. [37]

An interesting and ongoing discussion is the use of stemless implant fixation with sleeves in rTKA. In vitro data shows a dominant fixation of the sleeve and limited increase of stability through the addition of a stem. Possible advantages may be reduced implant length and cost, more natural bone formation and overcoming the problem of malalignment in bowed bones. [38] Clinical data shows promising short-term results with stemless sleeves. [39, 40] However, more data especially on reduced bone quality, increased BMI and the increased force transmission in rotating-hinge prosthesis are required. [38]

**Fig. 9:**
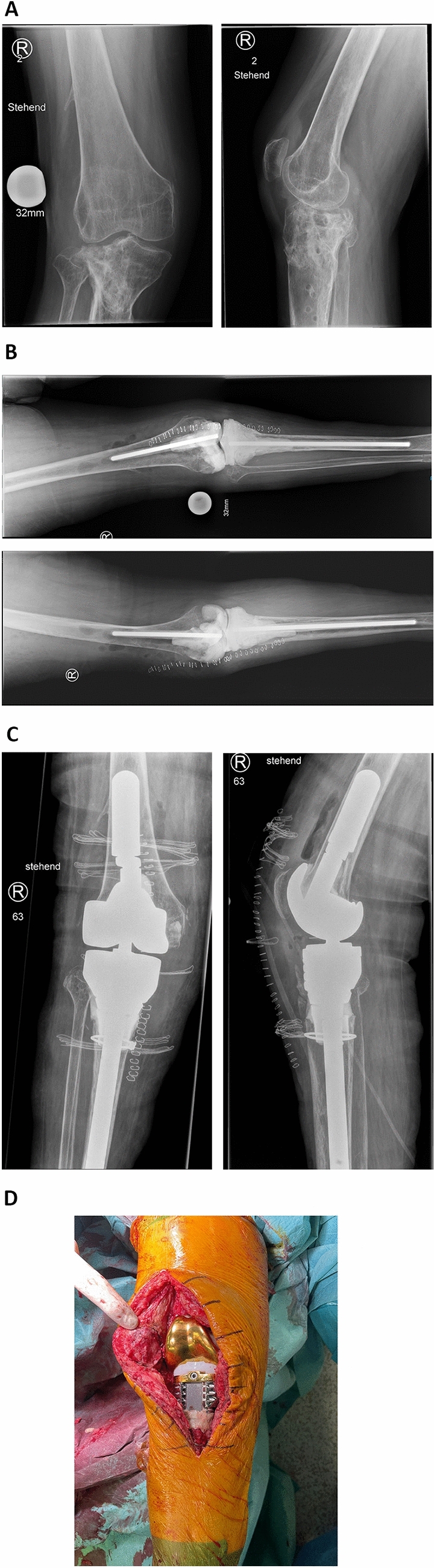
Case 3 shows a 65 year old female patient presenting with an infected pseudarthrosis and secondary osteoarthritis of the knee joint after suffering a tibial head fracture (AO 41C3) and a open reduction and internal fixation 2 years prior. The plate osteosynthesis had been removed prior to the first presentation in our out-patient clinic. We planned a removal of the osteitic bone, the collection of multiple deep tissue samples for histopathologic and microbiologic evaluation and the implantation of an articulating custom-made spacer. **A** Show the preoperative radiographs upon first presentation in our out-patient clinic. Clinically the patient experienced a multidirectional instability (KDC B) and a valgisation of the leg axis. The arrows mark the osteitic bone. **B** Postoperative radiographs after the first operation showing an articulating custom-made spacer with a severe erosion of the tibial bone stock (KDC 4) and cement-augmented defects of the femoral condyles (KDC 2). A thin bone layer at the insertion of the patella tendon could be preserved. **C** Postoperative radiographs after the reimplantation in an anterior–posterior (left) and lateral view (right). The femoral component could be well fixated through distal und posterior metallic augmentation and PMMA-form fitting (first zone) and a diaphyseal anchoring uncemented stem (second zone). The diaphyseal destruction was augmented using a macro-porous cone. A cerclage wire has been added prior to impaction due to poor bone quality and to prevent a fissure. The metaphyseal bone was completely replaced by a large metal augment featuring attachment points for the conservation of the extension apparatus (first zone). A long uncemented stem was added to achieve further fixation in the uncompromised part of the diaphyseal bone (second zone). **D** Clinical image in the frontal view before attaching the residual insertion of the patella tendon to the augment

In type 3 defects the metaphyseal bone loss presents with increases severity and an uncontained defect of the cortical bone. Metaphyseal cones are not directly connected to the prosthesis, but rather augment the host bone before the TKA is implanted. While partially overlapping in indication with metaphyseal sleeves, cones are usually reserved for severely deficient metaphyseal bone stock (KDC 3). They are macroporous, osteoconductive and have a similar elasticity to cortical bone. [41] Favorable results have been reported in the literature. [42, 43] Once the cone has achieved a sufficient press-fit bridging the metaphyseal defect, the prosthesis will be cemented in the center of the cone (see Fig. 9).

The utilization of structural bulk allografts for both KDC 2 and 3 defects has been described but is not recommended by the authors. While bulk allografts offer the potential of defect downsizing, they have a risk of nonunion, resorption and collapse leading to structural instability. [44] Especially in larger defects (AORI 2 and 3), bulk allografts show a significantly higher rate of loosening and overall failure when compared to porous metal augmentation. [45]

Diaphyseal stems offer the facilitation of load transfer and distribution of stress in cases of rTKA with compromised epiphyseal/metaphyseal bone loss. Adhering to the principle of zonal fixation, the addition of a diaphyseal anchoring stem serves as the required secondary zonal fixation. [6] Stems may be cemented, hybrid or uncemented and can be part of a monobloc prosthesis or modular. Hybrid fixation describes the combination of an uncemented stem and epiphyseal/metaphyseal cement placement in the bone-implant interface. Multiple publications have investigated all three techniques with similar good results making choice of stem fixation controversial and largely dependent on the surgeon’s preference. [42, 46] KDC type 4 defects extend into the diaphyseal bone and need to be bridged with diaphyseal anchoring stems of a sufficient length. Additional fixation using cerclage wires, strut grafts and/or distal/proximal locking screws maybe required. Depending on the residual bone stock of the epiphyseal/metaphyseal bone in type 4 defects, a reconstruction may not be feasible and a megaprosthesis can be implanted. Traditionally introduced for the treatment of tumor lesions, megaprosthesis have become more common in cases of complex bone loss in rTKA. Depending on the extent of the bony destruction, condylar replacement or even distal femoral/tibial/replacement in varying length can be necessary. Using megaprosthesis in rTKA is associated with reduced functionality, high risk of infection and reduced outcome. [47] Conservation or reattachment of the tibial insertion of the patellar tendon can be challenging.

Ligamentary instability in cases of rTKA is a common complication. Possible causes may be bone deficiencies with an altered joint line, **excessive varus or valgus alignment,** ligamentary and soft tissue deficiency or a combination of both. KDC type A describe a reconstructable instability. The patient has functioning ligaments and soft tissue and the instability is caused by alteration of the bony anatomy. Reasons may be implant malpositioning or uncontained defects resulting in structural instability. The aim of the rTKA should be the anatomically correct reconstruction of the joint line which will solve the instability in KDC A cases. KDC B cases may also exhibit structural instabilities, but are characterized by an insufficiency of the collateral ligaments and/or the posterior cruciate ligament (if a cruciate retaining prosthesis was used). An insufficiency of the posterior cruciate ligament may also be due to the use of a posterior stabilizing total knee arthroplasty. While anatomic reconstruction of the joint line remains crucial, an additional form of constraint (mechanical substitution of ligament function) is required. As defined by Morgan-Jones and Graichen, the goal should be 'minimal constraint and maximum balance'. Balance refers to appropriate tension in all planes of movement, which can be achieved by anatomic reconstruction of the original bony surface and joint line. [48] The type of constrainment can vary from varus-valgus constrains up to rotating-hinge design. KDC C is the most severe ligamentary dysfunction displaying an insufficiency of the extensor mechanism resulting in an inability to extend the knee joint. Reconstruction of the extensor mechanism is challenging and has a significant risk of overall failure and infection. Different surgical techniques are described using allograft or synthetic reconstruction. In a recent meta-analysis of 30 studies, the group around Deren et al. could not find any significant difference in overall failures, infections, functional outcomes, or revision constructions between allografts and synthetic material and reported an overall high rate of failure (23.2%). [49] If an extensor repair is not possible, the only feasible option is an arthrodesis. As a limb salvage arthrodesis shows a pain reduction and preservation of quality of life and everyday mobility. [50] The complete therapeutic algorithm is provided in Table 2.

**Table 2:** Treatment options according to defect type

KDC defect type	Treatment option
1	PMMA, allograft or augment + Additional support with (un-)cemented stem
2	Augment, sleeve or cone + Additional support with (un-)cemented stem
3	Sleeve or cone + Additional support with (un-)cemented stem
4	Briding of the diaphyseal defect with (un-)cemented stem + Additional support (cerclage wires, strut grafts, distal locking if necessary) + Treatment of metaphysis according to 2/3 or Megaprosthesis
A	Anatomical reconstruction of joint line
B	Anatomical reconstruction of joint line + Adequate constrainment (PS/VVC/Rotating-hinge)
C	Biological/synthetic reconstruction + Anatomical reconstruction of joint line + Rotating-hinge design or Arthrodesis

This publication has the following limitations. Creating a classification system always tends to be a compromise between perfect morphological defect description and simplicity. The authors decided to only focus on differences between defects which create an actual change in therapeutic protocol. Therefore, defect descriptions are simplified and may not extensively describe every defect that could be encountered. The surgeon must remain vigilant and may need to abstract certain defect morphologies according to the involvement of the main structural components of the femur and tibia and alter the therapeutic algorithm accordingly.

While the intended use of the KDC extends to all established means of preoperative imaging as well as the intraoperative setting, it is important to note that this study only provides results applicable to the use of native, preoperative radiographs. While the reliability and reproducibility for other imaging techniques (e.g., CT scans or MRIs) is expected to be equally good, no data was collected to substantiate that claim.

If preoperative evaluation is performed with an implant still in place (e.g., one-stage aseptic/septic exchange), further structural damage to the bone stock due to implant removal must be anticipated and requires some clinical experience. The defect grading may need to be altered accordingly. The KDC grading of the intraoperative findings has been evaluated retrospectively and limits the evidence level of the acquired agreement with the preoperative radiographic grading. The available recorded preoperative data did not allow for an analysis of reliability of the grading of ligamentary insufficiency. Therefore, the presented publication does not provide evidence concerning the reliability of the ligamentary assessment. Prospective studies are at the time of publication on-going and will be published in the near future.

## Conclusion

The Knee Defect Classification (KDC) is a reliable and reproducible classification system. Through its structured design it facilitates intuitive use and allows for consistent preoperative planning and intraoperative guidance. A therapeutic algorithm is provided based on a thorough review of the current literature in combination with expert opinion. The introduction of the KDC adds another major field of revision arthroplasty to the recently introduced and expanding collection of classification systems by the authors. Together with the Acetabular Defect Classification (ADC) and the Femoral Defect Classification (FDC), an integrated, didactically sound, and therapeutically-based classification system for revision arthroplasty of multiple joints has been established.

## Data Availability

The manuscript has no associated data.
